# Supplementary material for: The KRAS-Mutant Consensus Molecular Subtype 3 Reveals an Immunosuppressive Tumor Microenvironment in Colorectal Cancer
Source: Cancers (Basel). 2023 Feb 8;15(4):1098. doi: 10.3390/cancers15041098 (PMC9953921; doi:10.3390/cancers15041098)
Supplement: Supplementary file 1 [file cancers-15-01098-s001.zip › Supplementary Materials/Supplementary Table S2 .docx]

Supplemental Table S2. The enrichment analysis of top ten canonical pathways correlating with immune and TME pathways for 92 DEGs of *KRAS*^mut^ by IPA analysis

| Name | Total genes | Gene enrichment | Percentage (%) | *P* value |
| --- | --- | --- | --- | --- |
| Regulation of the epithelial mesenchymal transition (EMT) by growth factors pathway | 192 | 12 | 6.2 | 1.08×10^-11^ |
| HIF1 signaling | 208 | 12 | 5.8 | 2.76×10^-11^ |
| MSP-RON signaling in cancer cells pathway | 140 | 10 | 7.1 | 1.68×10^-10^ |
| IL-15 production | 123 | 9 | 7.3 | 1.16×10^-9^ |
| TGFβ signaling | 96 | 8 | 8.3 | 3.60×10^-9^ |
| Regulation of the EMT pathway | 195 | 10 | 5.1 | 4.26×10^-9^ |
| Tumor microenvironment (TME) pathway | 179 | 8 | 4.5 | 4.67×10^-7^ |
| JAK/STAT signaling | 82 | 6 | 7.3 | 7.71×10^-7^ |
| PI3K/AKT signaling | 199 | 7 | 3.5 | 1.22×10^-5^ |
| STAT3 pathway | 135 | 6 | 4.4 | 1.41×10^-5^ |

Fisher’s exact test was used to calculate the probabilities between input gene set with the canonical pathway.
