# Supplementary material for: The KRAS-Mutant Consensus Molecular Subtype 3 Reveals an Immunosuppressive Tumor Microenvironment in Colorectal Cancer
Source: Cancers (Basel). 2023 Feb 8;15(4):1098. doi: 10.3390/cancers15041098 (PMC9953921; doi:10.3390/cancers15041098)

Supplemental Figure S1. The IPA pathway analysis of 92 DEGs for *KRAS*^{mut} from the NanoString PanCancer progression panel reveals that the illustration map of TGFβ signaling pathway was activated

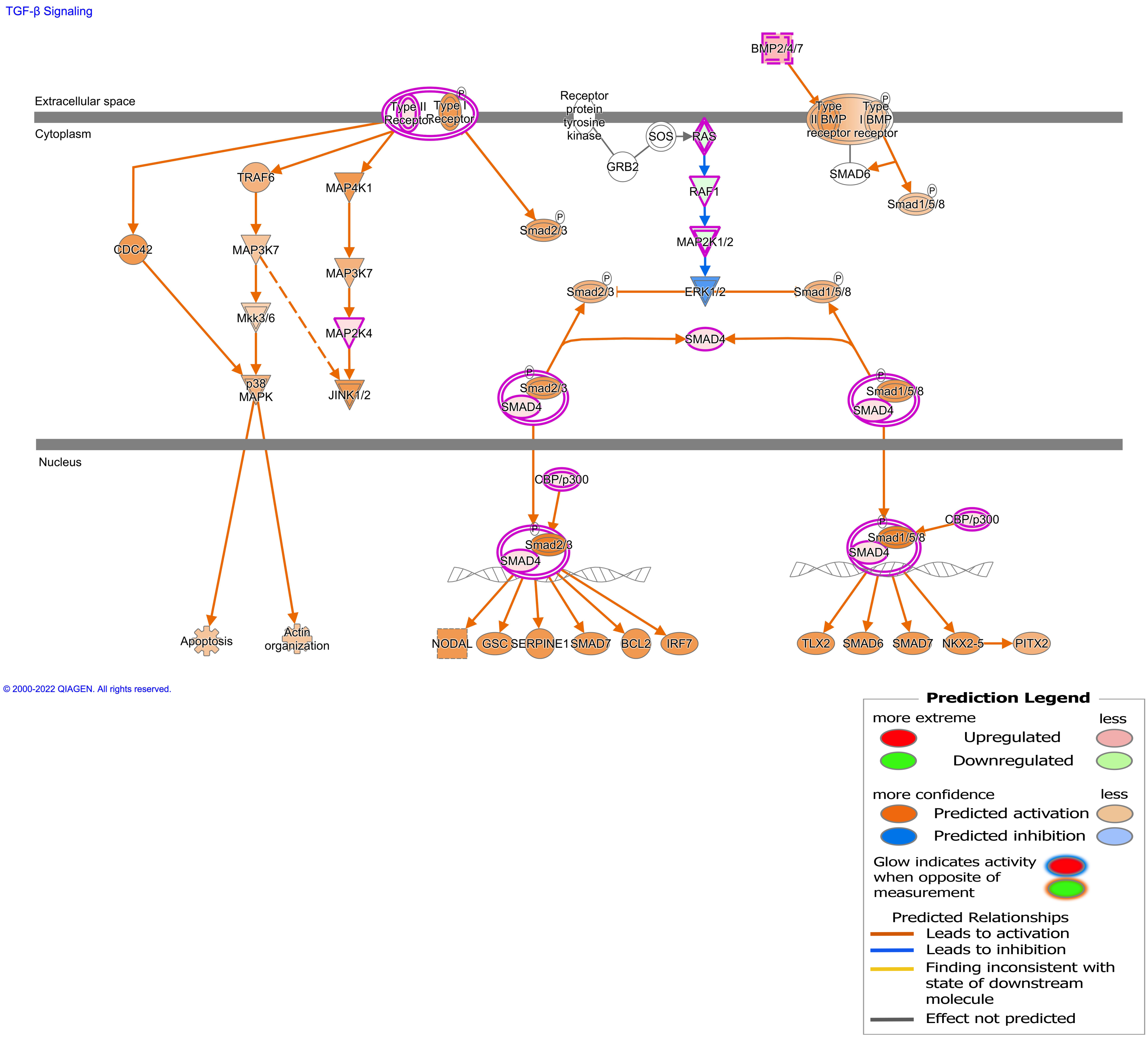

Supplement: Supplementary file 1 [file cancers-15-01098-s001.zip › Supplementary Materials/Supplementary Figure S1.pdf]
