# Supplementary material for: The KRAS-Mutant Consensus Molecular Subtype 3 Reveals an Immunosuppressive Tumor Microenvironment in Colorectal Cancer
Source: Cancers (Basel). 2023 Feb 8;15(4):1098. doi: 10.3390/cancers15041098 (PMC9953921; doi:10.3390/cancers15041098)

Supplemental Figure S2. The IPA pathway analysis of 15 DEGs for TME cancer regions and 37 DEGs for cancer from digital spatial profiling (DSP) GeoMx immune pathway panel reveals that DEGs are enriched in the TME canonical pathway region

Tumor microenvironment (TME) *KRAS*^{mut}

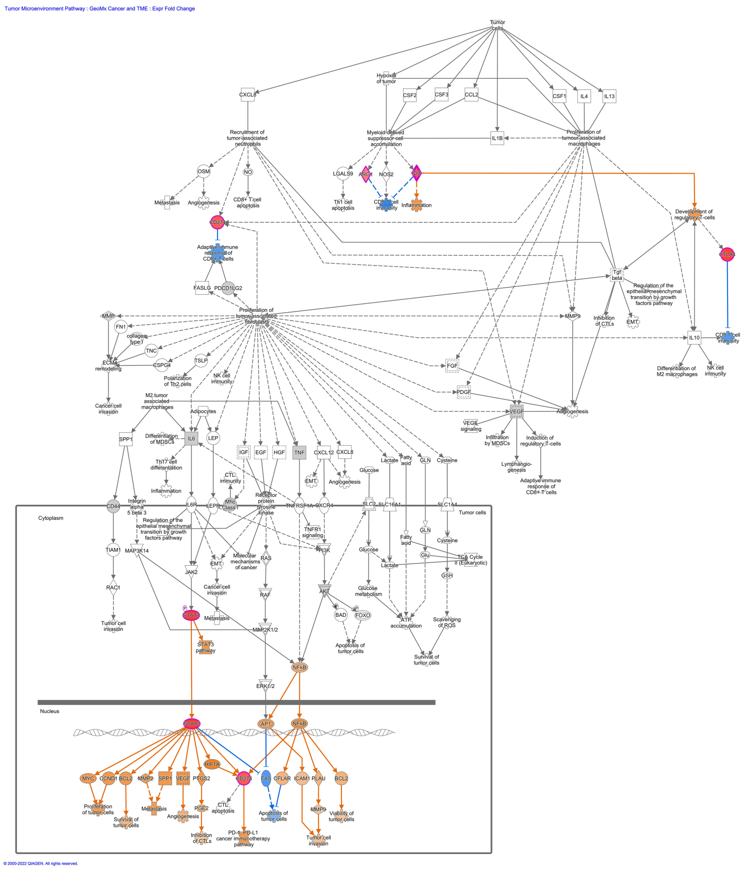

Cancer *KRAS*^{mut}

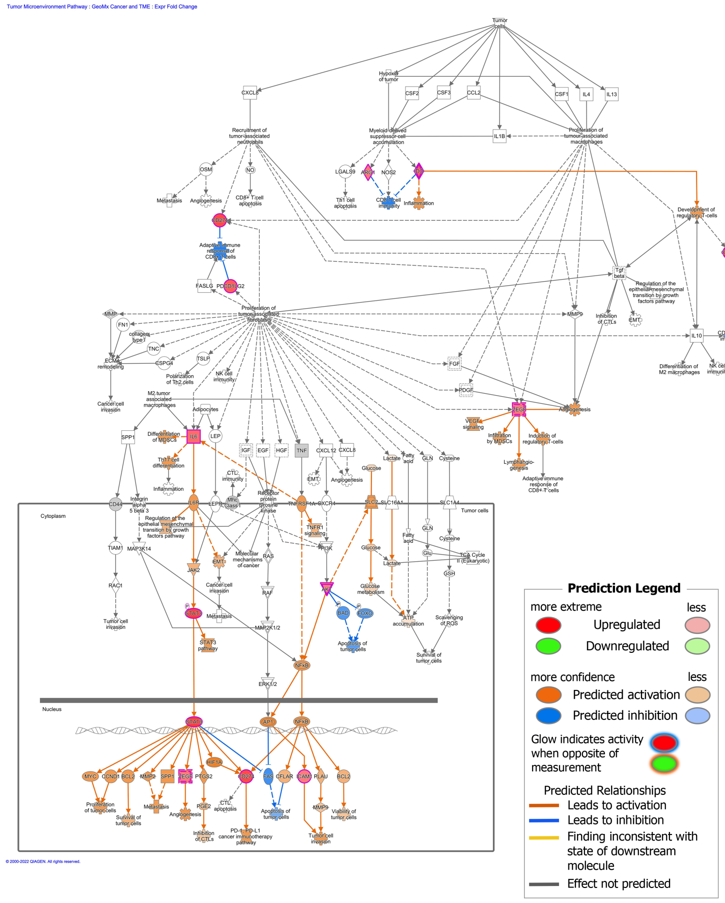

Supplement: Supplementary file 1 [file cancers-15-01098-s001.zip › Supplementary Materials/Supplementary Figure S2.pdf]
