# Supplementary material for: The KRAS-Mutant Consensus Molecular Subtype 3 Reveals an Immunosuppressive Tumor Microenvironment in Colorectal Cancer
Source: Cancers (Basel). 2023 Feb 8;15(4):1098. doi: 10.3390/cancers15041098 (PMC9953921; doi:10.3390/cancers15041098)
Supplement: Supplementary file 1 [file cancers-15-01098-s001.zip › Supplementary Materials/Supplementary Table S1.docx]

Supplemental Table S1. The significant 92 DEGs of *KRAS*^mut^

| Symbol | GeneID | Description | log2FoldChange | *P* value | *P* adj |
| --- | --- | --- | --- | --- | --- |
| FREM1 | 158326 | FRAS1 related extracellular matrix 1 | 2.27225 | 0.00000338 | 0.00218 |
| CCL8 | 6355 | chemokine (C-C motif) ligand 8 | -1.9799 | 0.0000802 | 0.02592 |
| ERMP1 | 79956 | endoplasmic reticulum metallopeptidase 1 | 0.422191 | 0.00014 | 0.03074 |
| MTA1 | 9112 | metastasis associated 1 | 0.891176 | 0.00055 | 0.07628 |
| CDKN1A | 1026 | cyclin-dependent kinase inhibitor 1A (p21_ Cip1) | 0.691025 | 0.00059 | 0.07628 |
| EPHA1 | 2041 | EPH receptor A1 | 0.668922 | 0.00111 | 0.09851 |
| RPS27A | 6233 | ribosomal protein S27a | 0.327331 | 0.00121 | 0.09851 |
| TOM1L1 | 10040 | target of myb1 like 1 membrane trafficking protein | 0.318888 | 0.00122 | 0.09851 |
| F11R | 50848 | F11 receptor | 0.384116 | 0.00141 | 0.10145 |
| SH3YL1 | 26751 | SH3 and SYLF domain containing 1 | 0.46535 | 0.00213 | 0.11826 |
| TGFBR2 | 7048 | transforming growth factor_ beta receptor II (70/80kDa) | 0.324344 | 0.00233 | 0.11826 |
| HRAS | 3265 | Harvey rat sarcoma viral oncogene homolog | -0.319908 | 0.00252 | 0.11826 |
| TCF3 | 6929 | transcription factor 3 | -0.445749 | 0.00284 | 0.11826 |
| BMP4 | 652 | bone morphogenetic protein 4 | 0.777936 | 0.00288 | 0.11826 |
| HOXB3 | 3213 | homeobox B3 | 0.580254 | 0.00289 | 0.11826 |
| ID2 | 3398 | inhibitor of DNA binding 2_ dominant negative helix-loop-helix protein | 0.465438 | 0.00293 | 0.11826 |
| EGLN3 | 112399 | egl-9 family hypoxia-inducible factor 3 | 0.82473 | 0.00338 | 0.12843 |
| SHB | 6461 | Src homology 2 domain containing adaptor protein B | 0.616706 | 0.0036 | 0.12902 |
| HOXA5 | 3202 | homeobox A5 | 0.884431 | 0.00459 | 0.1562 |
| MGAT5 | 4249 | mannosyl (alpha-1_6-)-glycoprotein beta-1_6-N-acetyl-glucosaminyltransferase | 0.281076 | 0.00561 | 0.18111 |
| RAF1 | 5894 | Raf-1 proto-oncogene_ serine/threonine kinase | -0.239213 | 0.00612 | 0.18834 |
| GTF2I | 2969 | general transcription factor IIi | 0.210334 | 0.00683 | 0.19001 |
| ECM1 | 1893 | extracellular matrix protein 1 | 0.700641 | 0.00701 | 0.19001 |
| EPS8L1 | 54869 | EPS8-like 1 | 0.595025 | 0.00734 | 0.19001 |
| VEZF1 | 7716 | vascular endothelial zinc finger 1 | 0.163541 | 0.0074 | 0.19001 |
| VWA2 | 340706 | von Willebrand factor A domain containing 2 | 0.861491 | 0.00765 | 0.19001 |
| SLC44A4 | 80736 | solute carrier family 44_ member 4 | 0.954052 | 0.00829 | 0.19746 |
| TGFBI | 7045 | transforming growth factor_ beta-induced_ 68kDa | 0.540614 | 0.00856 | 0.19746 |
| SMAD4 | 4089 | SMAD family member 4 | 0.292673 | 0.00968 | 0.21557 |
| CD46 | 4179 | CD46 molecule_ complement regulatory protein | 0.328227 | 0.01183 | 0.25483 |
| ZC3H12A | 80149 | zinc finger CCCH-type containing 12A | 0.49864 | 0.01245 | 0.25946 |
| DLL4 | 54567 | delta-like 4 (Drosophila) | -0.384979 | 0.01317 | 0.26209 |
| CD2AP | 23607 | CD2-associated protein | 0.238855 | 0.01339 | 0.26209 |
| CTSH | 1512 | cathepsin H | -0.437967 | 0.01456 | 0.2724 |
| GPI | 2821 | glucose-6-phosphate isomerase | -0.242596 | 0.0148 | 0.2724 |
| TLR4 | 7099 | toll-like receptor 4 | 0.556912 | 0.01556 | 0.2724 |
| MAP2K4 | 6416 | mitogen-activated protein kinase kinase 4 | 0.24179 | 0.01587 | 0.2724 |
| TJP2 | 9414 | tight junction protein 2 | 0.227549 | 0.01626 | 0.2724 |
| EPHB4 | 2050 | EPH receptor B4 | 0.292701 | 0.01682 | 0.2724 |
| ICAM1 | 3383 | intercellular adhesion molecule 1 | -0.584846 | 0.01708 | 0.2724 |
| NRCAM | 4897 | neuronal cell adhesion molecule | 1.25544 | 0.01729 | 0.2724 |
| CXCR3 | 2833 | chemokine (C-X-C motif) receptor 3 | -0.461641 | 0.01796 | 0.27257 |
| ERBB2 | 2064 | erb-b2 receptor tyrosine kinase 2 | 0.342095 | 0.01815 | 0.27257 |
| ID4 | 3400 | inhibitor of DNA binding 4_ dominant negative helix-loop-helix protein | 0.50917 | 0.01922 | 0.27257 |
| SOX9 | 6662 | SRY (sex determining region Y)-box 9 | 0.343617 | 0.02014 | 0.27257 |
| TACSTD2 | 4070 | tumor-associated calcium signal transducer 2 | 1.17007 | 0.02014 | 0.27257 |
| ITGB4 | 3691 | integrin_ beta 4 | 0.393606 | 0.02019 | 0.27257 |
| PLAUR | 5329 | plasminogen activator_ urokinase receptor | -0.743474 | 0.02057 | 0.27257 |
| EGLN2 | 112398 | egl-9 family hypoxia-inducible factor 2 | -0.187139 | 0.02068 | 0.27257 |
| HIPK2 | 28996 | homeodomain interacting protein kinase 2 | 0.306116 | 0.02166 | 0.27989 |
| STAT1 | 6772 | signal transducer and activator of transcription 1_ 91kDa | -0.301256 | 0.02485 | 0.30065 |
| COL4A2 | 1284 | collagen_ type IV_ alpha 2 | -0.559897 | 0.02522 | 0.30065 |
| NFKB1 | 4790 | nuclear factor of kappa light polypeptide gene enhancer in B-cells 1 | 0.235591 | 0.0253 | 0.30065 |
| EGFL7 | 51162 | EGF-like-domain_ multiple 7 | -0.535529 | 0.02551 | 0.30065 |
| BRMS1 | 25855 | breast cancer metastasis suppressor 1 | -0.178018 | 0.0256 | 0.30065 |
| SPINK5 | 11005 | serine peptidase inhibitor_ Kazal type 5 | -0.911581 | 0.02726 | 0.30217 |
| CXCL10 | 3627 | chemokine (C-X-C motif) ligand 10 | -0.736119 | 0.0274 | 0.30217 |
| AGGF1 | 55109 | angiogenic factor with G patch and FHA domains 1 | 0.352957 | 0.02748 | 0.30217 |
| PDGFA | 5154 | platelet-derived growth factor alpha polypeptide | -0.426453 | 0.02801 | 0.30217 |
| MMP10 | 4319 | matrix metallopeptidase 10 | -1.06802 | 0.02807 | 0.30217 |
| ECSCR | 641700 | endothelial cell surface expressed chemotaxis and apoptosis regulator | -0.488759 | 0.02884 | 0.30541 |
| FGL2 | 10875 | fibrinogen-like 2 | -0.354308 | 0.0294 | 0.30632 |
| PLS1 | 5357 | plastin 1 | 0.363519 | 0.03039 | 0.30774 |
| ARAP2 | 116984 | ArfGAP with RhoGAP domain_ ankyrin repeat and PH domain 2 | 0.349102 | 0.03049 | 0.30774 |
| PKN1 | 5585 | protein kinase N1 | -0.291763 | 0.03101 | 0.30818 |
| PNPLA6 | 10908 | patatin-like phospholipase domain containing 6 | -0.228169 | 0.0315 | 0.30829 |
| RAB25 | 57111 | RAB25_ member RAS oncogene family | 0.32943 | 0.03278 | 0.31577 |
| NAA15 | 80155 | N(alpha)-acetyltransferase 15_ NatA auxiliary subunit | 0.206535 | 0.03375 | 0.31577 |
| ANPEP | 290 | alanyl (membrane) aminopeptidase | 1.08054 | 0.03414 | 0.31577 |
| CCR2 | 729230 | chemokine (C-C motif) receptor 2 | -0.545239 | 0.03422 | 0.31577 |
| LAMC2 | 3918 | laminin_ gamma 2 | 0.496817 | 0.03537 | 0.31769 |
| TNFRSF12A | 51330 | tumor necrosis factor receptor superfamily_ member 12A | -0.527156 | 0.03541 | 0.31769 |
| CREBBP | 1387 | CREB binding protein | 0.186804 | 0.03595 | 0.3181 |
| SKP1 | 6500 | S-phase kinase-associated protein 1 | 0.132676 | 0.03669 | 0.32023 |
| CBLC | 25974 | methylmalonic aciduria (cobalamin deficiency) cblC type_ with homocystinuria | 0.396019 | 0.03761 | 0.32023 |
| CALCRL | 10203 | calcitonin receptor-like | -0.477703 | 0.03806 | 0.32023 |
| TXNIP | 10628 | thioredoxin interacting protein | 0.405474 | 0.03829 | 0.32023 |
| MAP2K1 | 5604 | mitogen-activated protein kinase kinase 1 | -0.21133 | 0.03911 | 0.32023 |
| GJA5 | 2702 | gap junction protein_ alpha 5_ 40kDa | -0.580416 | 0.03932 | 0.32023 |
| PTK2B | 2185 | protein tyrosine kinase 2 beta | 0.335631 | 0.03999 | 0.32023 |
| IRF6 | 3664 | interferon regulatory factor 6 | 0.271096 | 0.04015 | 0.32023 |
| MMP12 | 4321 | matrix metallopeptidase 12 | -0.689478 | 0.04153 | 0.32709 |
| TYMP | 1890 | thymidine phosphorylase | -0.444194 | 0.04203 | 0.32709 |
| PPP2CB | 5516 | protein phosphatase 2_ catalytic subunit_ beta isozyme | 0.247638 | 0.04302 | 0.32878 |
| SCNN1A | 6337 | sodium channel_ non voltage gated 1 alpha subunit | 0.480889 | 0.0436 | 0.32878 |
| LLGL2 | 3993 | lethal giant larvae homolog 2 (Drosophila) | 0.264541 | 0.04377 | 0.32878 |
| CASP8 | 841 | caspase 8_ apoptosis-related cysteine peptidase | 0.1714 | 0.04536 | 0.33501 |
| CCL11 | 6356 | chemokine (C-C motif) ligand 11 | -0.592692 | 0.04564 | 0.33501 |
| SDC4 | 6385 | syndecan 4 | -0.316548 | 0.04777 | 0.3467 |
| MET | 4233 | MET proto-oncogene_ receptor tyrosine kinase | 0.328403 | 0.04881 | 0.3475 |
| SMC3 | 9126 | structural maintenance of chromosomes 3 | -0.138127 | 0.04895 | 0.3475 |
| CTNND1 | 1500 | catenin (cadherin-associated protein)_ delta 1 | 0.222437 | 0.04959 | 0.34818 |
| IGFBP7 | 3490 | insulin-like growth factor binding protein 7 | 2.27225 | 3.38E-06 | 0.00218 |
