# Supplementary material for: The KRAS-Mutant Consensus Molecular Subtype 3 Reveals an Immunosuppressive Tumor Microenvironment in Colorectal Cancer
Source: Cancers (Basel). 2023 Feb 8;15(4):1098. doi: 10.3390/cancers15041098 (PMC9953921; doi:10.3390/cancers15041098)
Supplement: Supplementary file 1 [file cancers-15-01098-s001.zip › Supplementary Materials/Supplementary Table S3.docx]

Supplemental Table S3. The enrichment analysis of top six canonical pathways of 37 DEGs in cancer and 15 DEGs TME regions of *KRAS*^mut^ by IPA analysis

| **Name** | **Total genes** | **Cancer region** | | | **TME region** | | |
| --- | --- | --- | --- | --- | --- | --- | --- |
|  |  | Gene enrichment | Percentage (%) | *P* value | Gene enrichment | Percentage (%) | *P* value |
| Th1 and Th2 activation pathway | 172 | 12 | 7.0 | 5.24×10^-19^ | 6 | 3.5 | 4.14×10^-10^ |
| Th1 pathway | 122 | 11 | 9.0 | 8.58×10^-18^ | 5 | 4.1 | 6.84×10^-9^ |
| Tumor microenvironment pathway | 179 | 10 | 5.6 | 4.24×10^-14^ | 5 | 2.8 | 4.69×10^-8^ |
| Pathogen induced cytokine storm signaling pathway | 372 | 11 | 3.0 | 2.00×10^-12^ | ND | ND | ND |
| Macrophage classical activation signaling pathway | 189 | 9 | 4.8 | 3.95×10^-12^ | 5 | 2.6 | 6.16×10^-8^ |
| T cell exhaustion signaling pathway | 567 | 12 | 2.1 | 8.16×10^-12^ | ND | ND | ND |
| Primary immunodeficiency signaling | 59 | ND | ND | ND | 3 | 5.1 | 5.44×10^-6^ |
| CD40 signaling | 67 | ND | ND | ND | 3 | 4.5 | 7.99×10^-6^ |

The Fisher’s exact test was used to calculate the probabilities between input gene set with the canonical pathway; ND, Not detection in top six canonical pathways
